# Primary cutaneous infection due to *Microascus cirrosus*: a case report

**DOI:** 10.1186/s12879-018-3535-5

**Published:** 2018-12-03

**Authors:** Lujuan Gao, Junjun Chen, Di Gao, Ming Li

**Affiliations:** Department of Dermatology, Zhongshan Hospital Fudan University, No.180 Fenglin Road, Xuhui District, Shanghai, 200032 China

**Keywords:** Cutaneous, *Microascus cirrosus*, Itraconazole, *Scopulariopsis*

## Abstract

**Background:**

*Microascus cirrosus,* the teleomorph of *Scopulariopsis* spp., is a saprobic species with a worldwide distribution and rarely causes human infection. In the present paper, we present the first case of primary cutaneous *M. cirrosus* infection in a Chinese female.

**Case presentation:**

A 17-year-old female presented with tender ulceration on her left ankle for three months. Histology revealed multiple branching, septate hyphae and moniliform fungal elements in the dermis. Tissue culture grew *M. cirrosus*, the teleomorph of *Scopulariopsis* spp., characterized by intercalary and ballooned, chlamydospore-like structures, annellidic and ampulliform conidiogenous cells along with truncated, bullet-shaped, smooth conidia and globose perithecial ascomata with cylindrical necks. Further molecular sequencing confirmed the identification. A diagnosis of primary cutaneous infection due to *M. cirrosus* was made. Treatment with itraconazole 200 mg per day for 10 weeks achieved significant improvement of the skin lesions.

**Conclusions:**

This case of uncommon mycotic cutaneous infection highlights the importance of mycological examination that help to recognize rare pathogenic fungi.

## Background

The genera *Microascus* and *Scopulariopsis* comprise species that commonly isolated from soil, air, decaying plant material, dung and moist indoor environments [1]. Previously, *Scopulariopsis* and *Microascus* species have not been considered as common human pathogenic fungi. However, recently the number of cases caused by these organisms has been on the rise. Among the genera *Microascus* and *Scopulariopsis*, *M. cirrosus* is an uncommon opportunistic fungi, which has been reported to cause superficial infections of toe nail, and systemic infection [2–5]. To our knowledge, primary cutaneous infection due to *M. cirrosus* has not been reported yet. Here, we report the first case of primary cutaneous infection of *M. cirrosus* that responded well to oral itraconazole.

## Case presentation

**Fig. 1:**
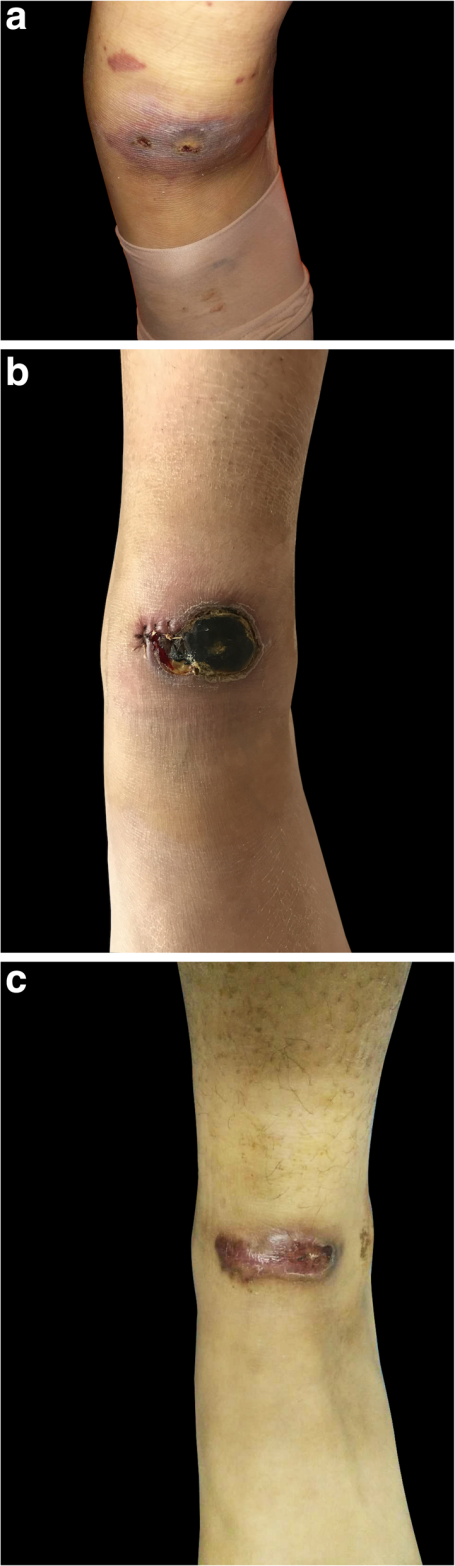
Clinical appearance. **a** Indurated erythema with central dusky necrosis at early stage. **b** Demarcated ulceration of 3.5 cm*2.5 cm, covered with thick black crust and purulent discharge. **c** Complete resolution of the ulceration with residual scar

A 17-year-old otherwise healthy Chinese female presented with tender skin lesion on her left ankle for almost three months. The lesion initially presented as an indurated erythema with central dusky necrosis, mimicking insect bites (Fig. 1a). She didn’t recall any evident history of trauma. During the same period, she developed extensive petechiae on both lower extremities and was diagnosed as Henoch-Schӧnlein purpura in local hospital. Subsequently she was treated with systemic corticosteroids (maximum dosage 30 mg/d) for two months. While the petechiae subsided, the ankle erythema ulcerated with suppurative discharge. She was then admitted to our hospital in March, 2017. Physical examination revealed a 3.5 cm*2.5 cm demarcated ellipsoidal ulceration covered with thick black crust and purulent discharge (Fig.1b). Regional lymph nodes were not palpable. Except for these lesions, the girl was generally in good health.

**Fig. 2:**
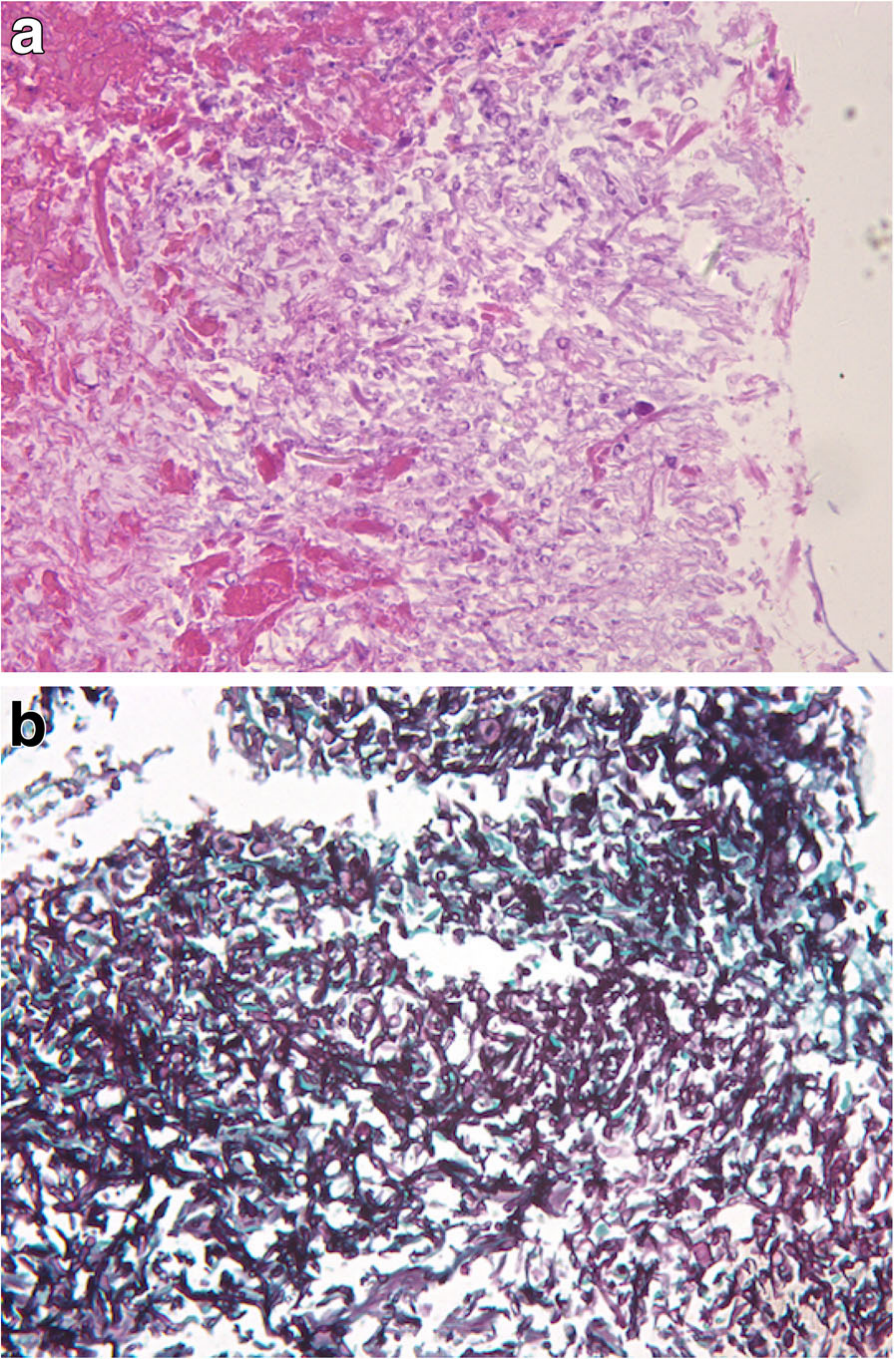
Histopathology findings. **a** Hematoxylin-eosin stained specimen revealed multiple branching, septate hyphae and moniliform fungal elements in the dermis (original magnification ✕ 400). **b** Gomori-Grocott methenamine silver stain was positive (original magnification ✕ 400)

Histological examination of biopsied tissue revealed multiple branching, septate hyphae and moniliform fungal elements in the dermis (Fig. 2a), which were positive with periodic acid–Schiff and Gomori-Grocott methenamine silver staining (Fig. 2b).

**Fig. 3:**
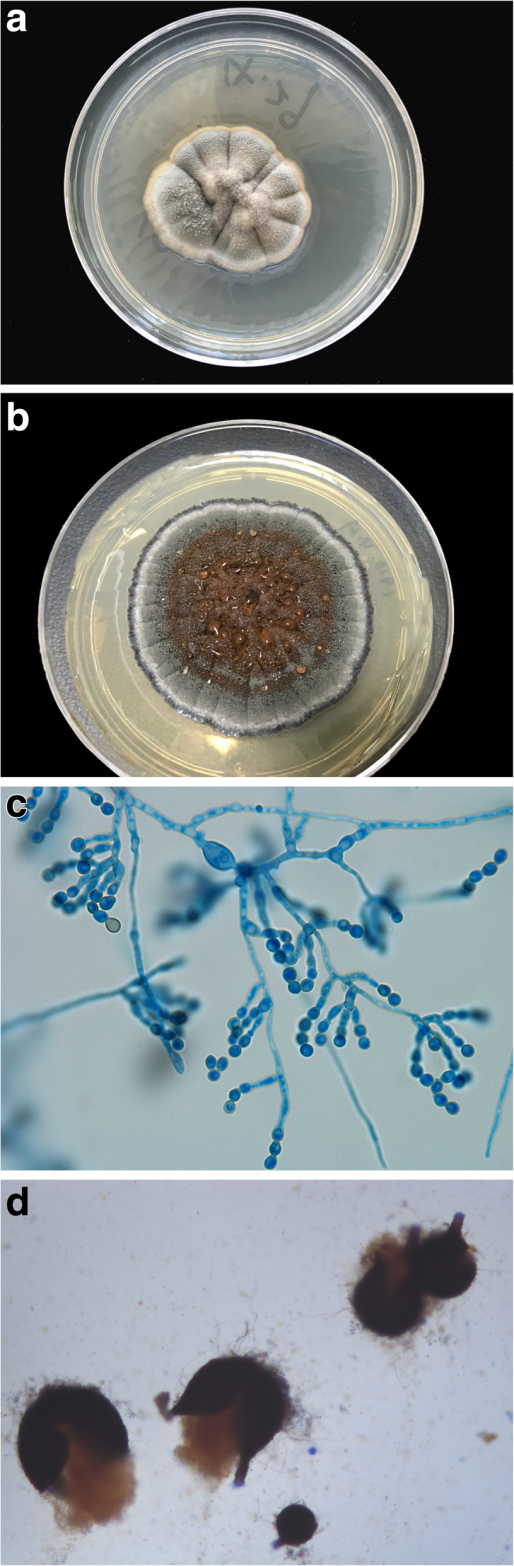
Mycological findings. **ab** Fungal culture incubated at 26 °C on Sabouraud’s dextrose agar grew white to grey and velvety colony at 4 weeks and turned brown at 8 weeks. **c** Lactophenol cotton blue stain of slides culture revealed filamentous and septate hyphae with intercalary and ballooned, chlamydospore-like structures, as well as annellidic and ampulliform conidiogenous cells along with truncated, bullet-shaped, smooth conidia (original magnification ✕ 1000). **d** Black fruits under microscopy showed globose perithecium with cylindrical necks, and a dark peridium, which are composed of dark brown cells (original magnification ✕ 200)

Tissue culture was performed and incubated at both 26 °C and 35 °C on Sabouraud’s dextrose agar (SDA) and yielded restricted, white to grey and velvety colony at 4 weeks (Fig. 3a). With extended incubation, the colony turned brownish at 8 weeks (Fig. 3b). Slides culture revealed filamentous and septate hyphae with intercalary and ballooned, chlamydospore-like structures, as well as annellidic and ampulliform conidiogenous cells along with truncated, bullet-shaped, smooth conidia (Fig. 3c), which resembled those found in *Scopulariopsis* species. Within 5 weeks, small black fruiting structures were growing on the surface of the colony, resulting in observance of globose perithecial ascomata with cylindrical necks, and a dark peridium (Fig. 3d). Fungal culture was identified as *M. cirrosus* based on the morphological features and confirmed by the molecular sequencing of internal transcribed spacer (ITS) region gene and β-tubulin gene. Comparison of the ITS (642 bp) (Genbank accession number MF450505) and β-tubulin (501 bp) (Genbank accession number MF455507) sequence with the Genbank database revealed 100% similarity with *M. cirrosus* reference strain CBS 116405 and CBS 115860, respectively.

In vitro susceptibility was tested according to the guidelines presented in document M38-A2 of the Clinical and Laboratory Standards Institute (CLSI) [6]. The minimum inhibitory concentration (MIC) was determined visually as the concentration that resulted in 100% inhibition [6]. The minimal effective concentration (MEC) endpoint was taken as the lowest concentration at which the visual growth pattern change from granular to filamentous growth was detected, microscopically seen as restricted hyphal growth. The results revealed that multiple antifungal agents were inactive against the isolate, with MIC value of 4 μg/ml for voriconazole (VRC), 8 μg/ml for caspofungin (CAS), >64 μg/ml for fluconazole (FLZ), >16 μg/ml for itraconazole (ITC), posaconazole (POS), amphotericin B (AMB) and terbinafine (TRB), and MEC value of > 16 μg/ml for micafungin (MCF). Due to the patient’s economic condition, itraconazole was applied. However, despite the discouraging result of in vitro susceptibilities, significant improvement of skin lesions was achieved after 10 weeks treatment with itraconazole 200 mg per day (Fig. 1c). No adverse event was reported.

## Discussion and conclusions

*M. cirrosus,* the teleomorph of *Scopulariopsis* spp., is a rare opportunistic fungus and commonly found in soil and decaying organic matter. Up to date, there are only six cases of *M. cirrosus* infections reported, including three cases of systemic infection [2, 3, 5], two case of onychomycosis [4] and one case of primary cutaneous infection (the present case) (summarized in Table 1). Patients with systemic infection all had underlying disease that compromise the immune system. The present case represents the first primary cutaneous infection of *M. cirrosus* in immunocompetent individual. The development of cutaneous infection of this patient may be associated with the rural area that she lives in. Despite she denied any evident history of trauma, outdoor activities might provide opportunity of minor wounds and inoculation of the pathogen. In addition, we suspected that the treatment with corticosteroids for Henoch-Schӧnlein purpura might had transiently suppressed the immunity, resulting in the persistent infection.

**Table 1:** Cases with *M. cirrosus* infection

No	Year	Sex	Age	Underlying disease	Involved sites	Treatment	Outcome	Reference
1	1992	F	56	–	Toenail	GSF, KTZ; Topical IMZ;	Resistant	[4]
		F	63	–	Toenail	GSF; Topical MCZ	Resistant	
2	1995	M	12	AML, BMT	Skin, Lung	AMB	Improvement in infection; Death due to AML relapse	[2]
3	2006	M	49	AML, BMT	Lung	AMB, VRC, TRB; Surgery;	Improvement in infection.	[3]
4	2018	F	60	Emphysema SOT	Tracheo-bronchia	AMB, VRC, TRB, CAS, Debridement	Cure	[5]
5	2018	F	17	–	Skin	ITC	Cure	–

Legend: *AML* Acute myelocytic leukemia, *BMT* *Bone* marrow transplantation, *SOT* Solid organ transplantation, *GSF* griseofulvin, *KTZ* ketoconazole, *IMZ* imidazole, *MCZ* miconazole

Due to the rarity of the infection, the appropriate antifungal regimen for *M. cirrosus* infection has yet to be defined. Early cases of toenail infections showed resistant to systemic treatment with griseofulvin (GSF), ketoconazole (KTZ), and topical application of imidazole (IMZ), miconazole (MCZ) [4]. However, recent cases with systemic *M. cirrosus* infection all showed improvement with debridement and antifungal chemotherapy of amphotericin B alone or combined with voriconazole, terbinafine, and caspofungin, as shown in Table 1 [2, 3, 5]. Although in vitro susceptibility testing in this case revealed that *M. cirrosus* was resistant to a variety of antifungals, the patient achieved favorable effects with itraconazole, suggesting that there is an inconsistency between in vitro *s*usceptibilities and in vivo antifungal effects.

In conclusion, although infection with *M. cirrosus* is a rare event, clinicians and microbiologists should be aware of the potential of *M. cirrosus* infection in both immunocompromised and immunocompetent individuals. Thorough mycological examination helps to recognize this rare pathogenic fungi, achieve an accurate diagnosis and initiate prompt antifungal treatments.

## Abbreviations

AMB: Amphotericin B
AML: Acute myelocytic leukemia
BMT: BMT Bone marrow transplantation
CAS: Caspofungin
CLSI: Clinical and Laboratory Standards Institute
FLZ: Fluconazole
GSF: Griseofulvin
IMZ: Imidazole
ITC: Itraconazole
ITS: Internal transcribed spacer
KTZ: Ketoconazole
MCF: Micafungin
MCZ: Miconazole
MEC: Minimal effective concentration
MIC: Minimum inhibitory concentration
POS: Posaconazole
SDA: Sabouraud’s dextrose agar
SOT: Solid organ
TRB: Terbinafine
VRC: Voriconazole
